# Lectin-Based Food Poisoning: A New Mechanism of Protein Toxicity

**DOI:** 10.1371/journal.pone.0000687

**Published:** 2007-08-01

**Authors:** Katsuya Miyake, Toru Tanaka, Paul L. McNeil

**Affiliations:** 1 Institute of Molecular Medicine and Genetics, Medical College of Georgia, Augusta, Georgia, United States of America; 2 Faculty of Pharmaceutical Sciences, Josai University, Sakado, Saitama, Japan; 3 Department of Cellular Biology and Anatomy, Medical College of Georgia, Augusta, Georgia, United States of America; University of California at Berkeley, United States of America

## Abstract

**Background:**

Ingestion of the lectins present in certain improperly cooked vegetables can result in acute GI tract distress, but the mechanism of toxicity is unknown. *In vivo*, gut epithelial cells are constantly exposed to mechanical and other stresses and consequently individual cells frequently experience plasma membrane disruptions. Repair of these cell surface disruptions allows the wounded cell to survive: failure results in necrotic cell death. Plasma membrane repair is mediated, in part, by an exocytotic event that adds a patch of internal membrane to the defect site. Lectins are known to inhibit exocytosis. We therefore tested the novel hypothesis that lectin toxicity is due to an inhibitory effect on plasma membrane repair.

**Methods and Findings:**

Repair of plasma membrane disruptions and exocytosis of mucus was assessed after treatment of cultured cell models and excised segments of the GI tract with lectins. Plasma membrane disruptions were produced by focal irradiation of individual cells, using a microscope-based laser, or by mechanical abrasion of multiple cells, using a syringe needle. Repair was then assessed by monitoring the cytosolic penetration of dyes incapable of crossing the intact plasma membrane. We found that cell surface-bound lectins potently inhibited plasma membrane repair, and the exocytosis of mucus that normally accompanies the repair response.

**Conclusions:**

Lectins potently inhibit plasma membrane repair, and hence are toxic to wounded cells. This represents a novel form of protein-based toxicity, one that, we propose, is the basis of plant lectin food poisoning.

## Introduction

Lectins are proteins that selectively bind carbohydrates and, importantly, the carbohydrate moieties of the glycoproteins that decorate the surface of most animal cells. They are found in a wide range of vegetables (29 out of 88 tested; [1]). Plant lectins that are not efficiently degraded by digestive enzymes, and that have an affinity for the surface of gut epithelial cells, such as those present in the Leguminosae family, can be poisonous [2]. Acute symptoms following ingestion include nausea, vomiting and diarrhea. Long-term intake in rodent models is characterized by increased cell turnover, gut hyperplasia and weight loss. Areas of epithelial cell necrosis and even zones of complete epithelial cell denudation are seen in biopsies of the stomach and intestine of mammals [3] and insects [4] fed plant lectins. Indeed, the plant lectin may function as a natural insecticide. Epithelial cell microvilli particularly are affected by lectin exposure, which initiates disruption and shedding of these membrane rich surface projections [5]. Confusingly, however, when cells are treated with lectins *in vitro*, even at very high doses, necrosis is not observed, though many other responses have been noted including mitogenesis [6], vacuole formation [7] and inhibition of exocytosis [8]. How then do lectins kill epithelial cells *in vivo*? This last mentioned effect, on exocytosis, suggested a possibility.

Epithelial cells lining the GI tract *in vivo*, unlike cells *in vitro*, are constantly exposed to mechanical stress and, consequently, frequently suffer plasma membrane disruptions [9]. However, cell death is not the only outcome of this type of injury: cells are capable of rapidly repairing and thereby surviving plasma membrane disruptions [10]. One key step of the repair mechanism, reviewed in [11], is exocytotic [12]. For large disruptions, this exocytotic reaction functions by adding a ‘patch’ of intracellular membrane to plasma membrane surrounding the disruption site [13]. Therefore, could the mechanism of lectin toxicity *in vivo* be due to an inhibitory effect on the exocytosis-based, constitutive membrane repair, and consequent death of wounded gut epithelial cells?

## Results and Discussion

**Figure 1:**
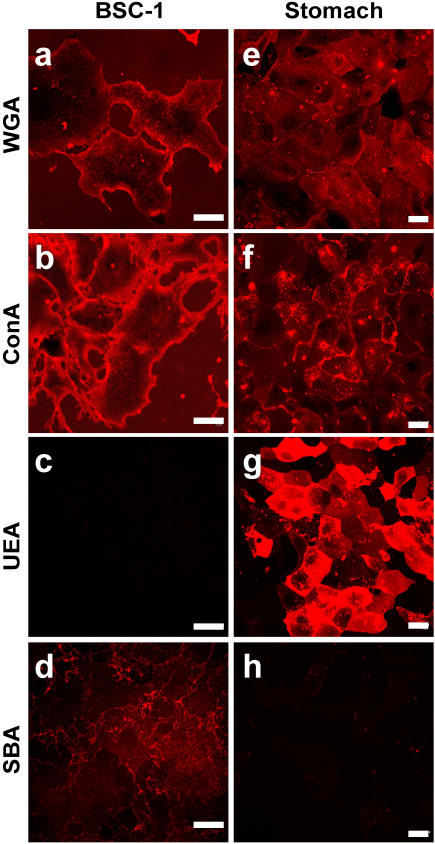
Certain lectins strongly stain the surface of live BS-C-1 cells and SSM cells. Living BS-C-1 cells are strongly stained with fluorescently labeled WGA (a) and ConA (b), but not with UEA (c) and SBA (d). SSM cells are stained with WGA (e), ConA (f) and, most intensely, with UEA (g) but not with SBA (h). Bars = 20 µm.

**Figure 2:**
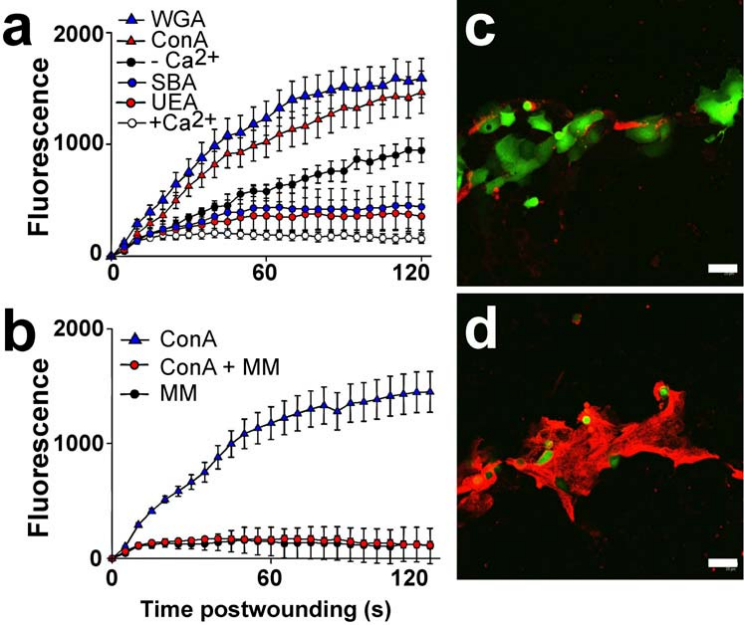
Lectins that bind to the surface of cells potently inhibit membrane repair. (a) Dye entry after laser-induced plasma membrane disruption was evaluated after incubation of cells in the indicated lectins. Dye exclusion from BS-C-1 cells was strikingly inhibited by the lectins Con A (red triangles) and WGA (blue triangles), but not by UEA (red circles) and SBA (blue circles). Positive controls (empty circles) were wounded in PBS containing 1.5 mM Ca^2+^, the vehicle for all lectins; negative controls (black circles) were wounded in PBS minus added Ca^2+^ (n = 18, ±S.E.M.).(b) Inhibition was prevented by the addition of competitive sugar, methyl-α-D-mannopyranoside (MM) in the case of ConA. (n = 18, ±S.E.M.). (c) Control, untreated SSM cell cultures were scratched to induce membrane disruptions along the injury site in the presence of FDx (10,000 MW, green), a marker of cells that suffer and repair a membrane disruption. They were then washed and FM 4-64 (red) was added (3 min later) to the medium: it acts under these conditions as a marker for cells that fail to reseal. The untreated cells along this scratch site are predominantly labeled with FDx, indicating successful repair of plasma membrane disruptions. (d) By contrast, WGA-treated cells lining this scratch site were predominantly labeled with FM 4-64.

To qualitatively determine lectin affinity for the plasma membrane of BS-C-1 cells, we incubated them in a panel of fluorescently labeled lectins. Wheat germ agglutinin (WGA) (Fig. 1a) and concanavalin A (ConA) (Fig. 1b), but not *Ulex europaeus* agglutinin I (UEA) (Fig. 1c) or soybean agglutinin (SBA) (Fig. 1d), strongly labeled the BS-C-1 cells. Repair following treatment with each of these lectins was then evaluated after creating a plasma membrane disruption with a laser. In this laser-based assay [14], we monitor intracellular staining with a normally membrane impermeant fluorescent dye, FM 1-43, as a function of time after injury. Repair blocks access of extracelluar dye to intracellular membrane and hence halts intracellular staining. In the presence of Ca^2+^, which is required for repair [12], no further dye uptake is measured 10–20 sec after the laser injury (Fig. 2a), and only a small ‘hot spot’ of intracellular staining, near the disruption site, is seen in video images (Movie S1). Resealing was rapidly (10–20 seconds) completed, as expected, under this physiological condition. When Ca^2+^ is omitted from the medium, dye uptake occurs over the entire time course of the typical measurement (Fig. 2a) and eventually all internal membrane will become stained (Movie S2). Resealing failed, as expected, in the absence of extracelluar Ca^2+^. Even more strikingly than in the case of omission of Ca^2+^, was the rapid, unrestricted influx of dye into ConA (Movie S3) and WGA (Movie S4) treated cells (Fig. 1a) after laser injury. By contrast, UEA (Movie S5) and SBA (Movie S6) did not have this effect (Fig. 2a). Inhibition of repair was rapidly achieved after lectin addition to cell cultures (<5 min incubation time, data not shown), dose-dependent (data not shown) and prevented by the addition of competitive sugar (Fig. 2b). Short-term treatment of cells with these lectins under the conditions used for laser wounding but in the absence of inducing plasma membrane disruptions did not, at all concentrations tested, adversely effect cell health (data not shown), either during the treatment (as assessed by FM 1–43 assay of dye entry) or after the treatment (as assessed by cell growth assay). Thus, those lectins observed to bind strongly to the BS-C-1 cell potently inhibited repair. Lectins were therefore toxic when a cell exposed to them experienced plasma membrane disruption.

In an independent test of this lectin effect, we wounded primary cultures of stomach surface mucous (SSM) epithelial cells by a mechanically-based, rather than laser-based, method, and assessed resealing also by an alternative method. First, as above, the surface plasma membrane of these cells were stained with a panel of fluorescent lectins to determine which bound to this cell type (Fig. 1 e–h). Only SBA failed to stain these cells. For wounding, the SSM cell culture substratum was scratched with a sharp implement: the cells lining the site denuded by the scratch suffer disruption injuries [15]. When this maneuver is carried out the presence of flurorescein dextran (FDx), a polar molecule of 10 kD, those cells that suffer and repair a disruption will trap this marker in their cytosol. Cells, on the other hand, that suffer and fail to repair a disruption do not trap the FDx (green fluorescence) but do become labeled with FM 4-64 (red fluorescence, separable from FITC and hence used instead of FM 1-43) added 5 min after the scratch is made. In untreated cells, FDx positive cells that successfully repaired disruptions were more common (Fig. 2c, green stain) than FM 4-64 positive cells (Fig. 2c, red stain) along scratch sites. The reverse pattern was observed after treatment with WGA (Fig. 2d), ConA (data not shown) or UEA (data not shown): FDx positive (green) cells were completely missing, only FM 4-64 positive (red) cells were observed. Treatment with the low affinity lectin, SBA (data not shown), resulted in a pattern similar to the control, e.g. a dense cluster of FDx positive cells along the injury site, and, comparatively few FM 4-64 positive cells. Thus, in a primary culture of GI tract epithelial cells, wounded by an alternative method, repair was also inhibited by cell surface binding lectins.

**Figure 3:**
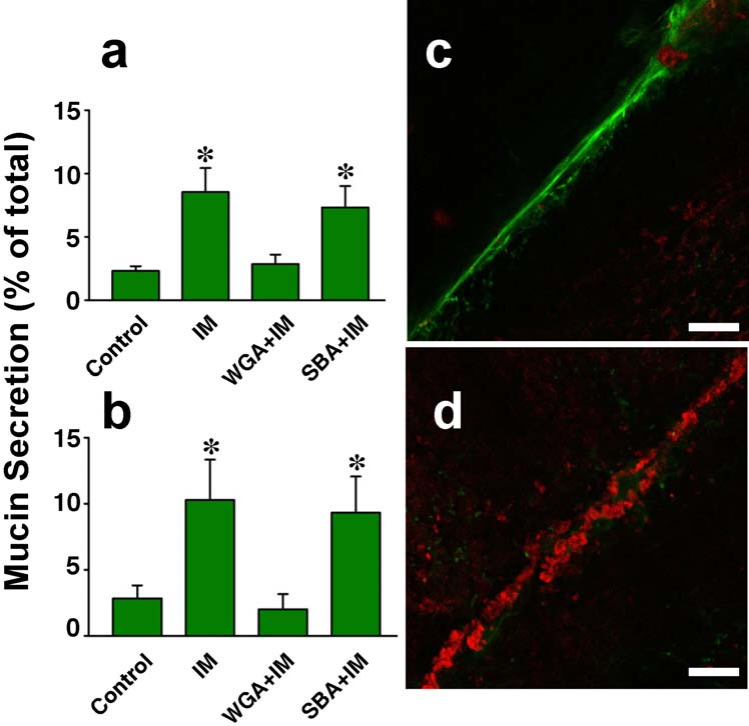
Lectins also potently inhibit mucus secretion. a,b) WGA blocks secretion of mucus by cultured SSM cells. Adherent cells were treated with WGA (WGA+IM) or SBA (SBA+IM) at a concentration of 100 µg/ml for 10 min and then incubated with a mucus-secretion inducing stimulus, Ca^2+^ ionophore (IM) (Ionomycin, 1 µM, 60 min). Control cells were not treated with ionophore or lectin (Control). The medium conditioned by these cells was then harvested and the content of mucus present quantitated using an ELLA assay employing either WGA (a) or UEA (b) as the mucus ligand. All treatments differing significantly from the control (Control) are decorated with an asterisk, n = 8, ±S.D., p≤0.001 (Panel a), ≤0.001 (Panel b), Kruskal-Wallis test. c) An excised, untreated control segment of mouse colon was scratched to induce plasma membrane disruptions in surface goblet cells and then incubated in FM 4-64 dye (to reveal dead cells that failed to reseal) and FITC-UEA (to reveal secreted mucus). Few dead cells (red staining) are observed along the scratch site but it is decorated with prominent mucus trail (green staining). d) In colon treated with WGA before scratching, dead cells (red staining) densely lined the scratch site but very little evidence of mucus secretion (green staining) is evident. Bars = 100 µm.

Since mucus secretion is evoked by membrane disruptions in mucus producing GI tract cells [16], we therefore asked if lectins also block secretion of this important lubricant. We found that, in cultures of SSM cells, lectin (WGA) potently blocked ionophore–induced mucus secretion (Fig. 3a,b). Moreover, when excised segments of mouse colon were scratched after treatment with WGA, the copious mucus secretion normally observed from the cells thus wounded (Fig. 3c, green stain) was dramatically reduced (Fig. 3d), while FM 4-64 positive dead cells were strikingly increased in density along the injury site (Fig. 3d, red stain). Thus, lectins also inhibited repair of resident gut epithelial cells, and additionally, this experiment shows, inhibited secretion of mucus by the cell subpopulation (goblet cells) that produces this lubricant.

The mechanism of lectin inhibition of membrane repair remains unclear. Exocytosis, which is required for membrane repair [11], is likely targeted by this class of toxin: previous studies showed that lectins can inhibit exocytosis [8], and we show here that, in particular, mucin exocytosis, which is coupled to repair in the cells we have studied, is potently inhibited by lectins. Moreover, we have found (data not shown) that inhibition of repair is rapid in onset (<1 min after addition to cell medium) and rapidly reversed by lectin washoff (<5 min after rinsing of cells with lectin-free medium containing competitive sugar). Therefore, inhibition is dependent on surface binding, and not on other, longer-term events (minutes–hours) such as lectin-induced cell vacuolation [7], which we have observed in the cells studied here (data not shown).

At least one form of muscular dystrophy is caused by a failure in the membrane repair mechanism [17]. We have shown here that binding of cell surface glycoproteins by lectins interferes with the exocytotic events associated with membrane repair (mucus secretion) and that lectins potently block repair. We here propose a second category of disease that can be explained as a failure in the plasma membrane repair mechanism. Lectins, we hypothesize, are toxic when present in the GI tract based on two, inter-related effects. First, resealing failure occurs within the general population of GI tract cells normally exposed to membrane disrupting levels of mechanical stress, leading to their necrosis. The second lectin-induced effect is exocytotic failure within the subpopulation of GI tract cells that normally secrete mucus, leading to a decrease in protective, lubricating mucus secretion and a consequent increase in the incidence of mechanically-induced membrane disruption events. Because lectins, based on the damage they do to the lining of the GI tract, and their hypertrophic effect, have been implicated in, respectively, celiac disease [18] and cancer [19], knowledge of this mechanism may have implications beyond a better understanding of food poisoning.

## Methods

### Cells

Stomach surface mucous (SSM cells) were isolated as previously described [16]. Briefly, the stomach was removed from an anesthetized rat, everted, inflated with Hank's buffer, and then immersed in dispase (50 U/ml, BD Biosciences) for 1 hour at 37°C. Surface mucous cells were dislodged by gentle trituration of the everted surface in Hank's containing 0.1% BSA and 0.5 mM EGTA, and were filtered through nylon mesh (150 gauge) prior to culture in 35 mm dishes on a collagen gel (Type 1, rat tail, BD Biosciences). Cells were used for experimentation 2–3 days later. BS-C-1 cells were cultured to confluence in DMEM medium containing 10% fetal bovine serum. In some cases, a segment of mouse (C57BL/6, male, 12 months) large intestine was removed, split longitudinally and washed thoroughly in PBS. It was then scratched with a 30-gauge syringe needle in PBS containing 1.5 mM Ca^2+^ or no added Ca^2+^.

### Surface Binding of Fluorescent Lectins

BS-C-1 or SSM cells were incubated in rhodamine conjugated lectins (Lectin Kit, Vector Laboratories, Inc.) dissolved in PBS at 20 µg/ml for 30 min at room temperature, washed with PBS 3 times, fixed in 4% freshly generated paraformaldehyde and then imaged on a Zeiss LSM510 Meta (excitation, 543 nm; emission, >565 nm)

### Laser Wounding Assay

Trypsinized cells seeded into 35 mm dishes were subject to laser wounding at 37 °C in the presence of FM 1-43 dye (2.5 µg/ml, Invitrogen), and dye uptake and total cell fluorescence due to FM 1-43 staining (excitation, 488 nm; emission, 520–590 nm) monitored by time lapse image acquisition and image analysis as previously described [14] on a multi-photon microscope (Zeiss LSM510 Meta) utilizing an infrared laser (Coherent). FM 1-43 is soluble in both water and lipid but fluoresces only in lipidic environments such as a cell membrane. Furthermore, while the dye freely enters and leaves the plasma membrane, it does not cross the bilayer. Therefore, initially, only the outer plasma membrane leaflet of an intact cell immersed in FM 1-43 fluoresces, though with time (minutes–hours) internal fluorescence can develop as a result of endocytosis. When, however, there is exposure of the relatively enormous pool of internal membrane to FM 1-43, as occurs after a cell suffers a plasma membrane disruption, cell fluorescence, due to staining of internal membrane, rapidly (seconds) increases until repair prevents further dye entry. Hence this event–membrane repair–is marked by a cessation of the increase in cell fluorescence initiated by the disruption.

### Scratch Wounding Assay

Cultured monolayers were scratched with a 30-gauge syringe needle in the presence of FDx (10 mg/ml) dissolved in PBS (containing 1.5 mM Ca^2+^), the culture was then washed 3 times with PBS, and FM 4-64 (2.5 µg/ml, in PBS) added. Finally, the culture was imaged confocally on a Zeiss LSM510 Meta (excitation, 488 nm; emission, >620 nm). Excised colon was scratched in PBS (containing 1.5 mM Ca^2+^) with a 30-gauge syringe needle, and, 3 min later, FITC-UEA (10 µg/ml, 10 min, Vector) was added. Finally, the colon was washed in PBS, FM 4-64 (2.5 µg/ml) added and the preparation immediately imaged confocally.

### Mucus Assay

Mucus release was quantified as previously described [16] using ELLA (enzyme linked lectin assay). Briefly, medium conditioned by adherent SSM cells was harvested by aspiration. For the ELLA, assay SBA (25 µg/ml, 3–5 hr, 21°C) was used to coat the bottoms of 96-well plates, which were then washed with buffer consisting of 0.1% BSA and 0.05% Tween20 in PBS. Following an overnight incubation with samples or standards (purified rat gastric mucin, 15–1000 ng/well), the samples were rinsed with wash buffer, and biotinylated WGA or UEA (1 µg/ml, 2 hr, 21°C) was added. Finally, biotin levels were detected, following further washing, using a standard kit (Vectastain ABC, Vector Laboratories Inc) and a plate reader (Spectra Max 250, Molecular Devices).

## Supporting Information

Movie S1 Movie (Quicktime) illustrating the typical successful resealing response of the BS-C-1 cell wounded by laser irradiation in the presence of extracellular Ca2+. Plasma membrane disruptions (6.7 µm diameter circular profile) were created (arrows mark the site) on 9 cells at the second frame of this, and all additional, time-lapse series (frame interval = 15 sec, 5 min total) of confocal images. FM 1-43 dye (green stain) begins immediately to enter through these disruptions, but, as evidenced by the limited ‘hot-spot’ of staining with this dye that develops only near the disruption site, rapidly ceases. Resealing was successful in all 9 of these cells.
(0.04 MB MOV)

Movie S2 Movie illustrating the typical failed resealing response of the BS-C-1 cell wounded by laser irradiation in the absence of extracellular Ca2+. FM 1-43 dye (green stain) begins immediately to enter through these disruptions, and this entry occurs throughout the time course of this movie. Resealing by all 7 of these cells fails.
(0.10 MB MOV)

Movie S3 Movie illustrating the typical failed resealing response of the BS-C-1 cell wounded by laser irradiation after ConA treatment. Note unrestricted influx of the FM 1-43 dye (green stain).
(0.27 MB MOV)

Movie S4 Movie illustrating the typical failed resealing response of the BS-C-1 cell wounded by laser irradiation after WGA treatment. Note unrestricted influx of the FM 1-43 dye (green stain).
(0.21 MB MOV)

Movie S5 Movie illustrating the typical successful resealing response of the BS-C-1 cell wounded by laser irradiation after UEA treatment. Note restriction of influx of the FM 1-43 dye (green stain).
(0.08 MB MOV)

Movie S6 Movie illustrating the typical successful resealing response of the BS-C-1 cell wounded by laser irradiation after SBA treatment. Note restriction of influx of the FM 1-43 dye (green stain).
(0.04 MB MOV)
